# Macrophages migrate in an activation-dependent manner to chemokines involved in neuroinflammation

**DOI:** 10.1186/1742-2094-11-23

**Published:** 2014-02-01

**Authors:** Daphne YS Vogel, Priscilla DAM Heijnen, Marjolein Breur, Helga E de Vries, Anton TJ Tool, Sandra Amor, Christine D Dijkstra

**Affiliations:** 1 Department of Molecular Cell Biology and Immunology, Neuroscience Campus Amsterdam VU University Medical Centre, MF J283, P.O. Box 7057, 1007, MB, Amsterdam, Netherlands; 2 Department of Pathology, VU University Medical Centre, Amsterdam, Netherlands; 3 Department of Blood Cell Research, Sanquin Research and Landsteiner Laboratory, Amsterdam, Netherlands; 4 Department of Neuroimmunology Unit, Blizard Institute, Barts and the London School of Medicine and Dentistry, Queen Mary University of London, London, UK

**Keywords:** Macrophages, migration, central nervous system, multiple sclerosis, macrophage activation

## Abstract

**Background:**

In neuroinflammatory diseases, macrophages can play a dual role in the process of tissue damage, depending on their activation status (M1 / M2). M1 macrophages are considered to exert damaging effects to neurons, whereas M2 macrophages are reported to aid regeneration and repair of neurons. Their migration within the central nervous system may be of critical importance in the final outcome of neurodegeneration in neuroinflammatory diseases e.g. multiple sclerosis (MS). To provide insight into this process, we examined the migratory capacity of human monocyte-derived M1 and M2 polarised macrophages towards chemoattractants, relevant for neuroinflammatory diseases like MS.

**Methods:**

Primary cultures of human monocyte-derived macrophages were exposed to interferon gamma and lipopolysaccharide (LPS) to evoke proinflammatory (M1) activation or IL-4 to evoke anti-inflammatory (M2) activation. In a TAXIScan assay, migration of M0, M1 and M2 towards chemoattractants was measured and quantified. Furthermore the adhesion capacity and the expression levels of integrins as well as chemokine receptors of M0, M1 and M2 were assessed. Alterations in cell morphology were analysed using fluorescent labelling of the cytoskeleton.

**Results:**

Significant differences were observed between M1 and M2 macrophages in the migration towards chemoattractants. We show that M2 macrophages migrated over longer distances towards CCL2, CCL5, CXCL10, CXCL12 and C1q compared to non-activated (M0) and M1 macrophages. No differences were observed in the adhesion of M0, M1 and M2 macrophages to multiple matrix components, nor in the expression of integrins and chemokine receptors. Significant changes were observed in the cytoskeleton organization upon stimulation with CCL2, M0, M1 and M2 macrophages adopt a spherical morphology and the cytoskeleton is rapidly rearranged. M0 and M2 macrophages are able to form filopodia, whereas M1 macrophages only adapt a spherical morphology.

**Conclusions:**

Together our results indicate that the alternative activation status of macrophages promotes their migratory properties to chemoattractants relevant for neuroinflammatory diseases like MS. Conversely, classically activated, proinflammatory macrophages have reduced migratory properties. Based on our results, we postulate that the activation status of the macrophage influences the capacity of the macrophages to rearrange their cytoskeleton. This is the first step in understanding how modulation of macrophage activation affects macrophage migration in neuroinflammatory diseases like MS.

## Introduction

Infiltration of circulating monocytes is a pathological hallmark of injury to the central nervous system (CNS). Once in the CNS, blood-derived macrophages are thought to contribute to tissue damage and repair in yet unidentified ways [1-3]. In the early phase of CNS injury, M1 (neurotoxic) macrophages were shown to be the first to appear at the site of injury followed by the appearance of M2 macrophages (axonal growth promoting) [1,2,4-7]. Paradoxically the role of macrophage polarisation in relation to their migration within the CNS tissue itself has been poorly studied. Recently, it was shown in mice that upon mechanical spinal cord injury, the recruitment of M1 and M2 macrophages in the CNS differs; M1 macrophages were found to derive from monocytes that entered the traumatized spinal cord dependent on Chemokine C-C motif ligand (CCL)2 through the adjacent spinal cord leptomeninges, whereas the M2 polarised cells were derived from monocytes that trafficked through the brain-ventricular choroid plexus [8]. Therefore, M2 macrophages are thought to migrate over longer distances. Previously we showed that murine bone marrow-derived M2 macrophages display enhanced motility compared to M1 macrophages towards CCL5 and Chemokine C-X-C motif ligand (CXCL)12 and that M2 cells are more attracted towards neuronal conditioned media, indicating that they may be attracted by neurons in the CNS [9]. Attraction of macrophages towards CNS injury may thus represent controlled recruitment of macrophages as necessary for repair.

Migration within the CNS towards sites of injury or inflammation is directed by chemokines. In the current study, we focus on chemokines, which are upregulated in neuroinflammatory diseases, such as multiple sclerosis (MS) [10-12]. In the inflammatory lesions, macrophages are the dominant cells. In general, chemokines are essential for attraction of macrophages towards the site of injury, for example, in MS lesions or stroke. CCL2 for example is produced by activated astrocytes and is known to play a key role in attracting monocytes over the blood-brain-barrier (BBB) [13,14]. Chemokines CCL5 and CXCL10 are known to be upregulated in the cerebral spinal fluid during relapses of MS and after stroke and CXCL12 is produced by reactive astrocytes in MS lesions [15-17]. Next to chemokines, the complement system may influence macrophage migration. C1q has been detected in blood vessel walls, astrocytes, along myelin and within macrophages/microglia in white matter MS lesions [18-20]. C1q is a known chemoattractant for monocytes and immature dendritic cells [21]. To our knowledge the role of C1q in macrophage migration is unclear. Here we studied the migration capacities of the pro- or anti-inflammatory macrophages towards CCL2, CCL5, CXCL10, CXCL12 and C1q.

Cell migration is closely regulated by cell polarisation due to dynamic changes of the cytoskeleton. Cytoskeletal changes of differently activated macrophages has been described in terms of morphology of the cells *in vitro*[22]. However, until now it has been unclear whether M1 and M2 human macrophages have different migration capabilities. We therefore addressed the question as to whether M1 and M2 macrophages have different directed migration capacities towards chemoattractants and chemokines which are known to play an important role in attraction of macrophages to support repair or cause damage in neuroinflammation in particular in MS [13,15,23-25].

## Materials and methods

### Macrophage isolation

Peripheral blood mononuclear cells (PBMCs) were isolated from buffy coats (Sanquin Blood Bank, Amsterdam, The Netherlands) obtained from healthy donors who gave informed consent for use of their blood for research purposes. Research was performed with the approval of the Medical Ethical Committee of the VU University Medical Center. PBMCs were isolated using Ficoll density gradient (Lymphoprep™, Axis-Shield, Oslo, Norway). Monocytes were isolated from PBMCs using anti-CD14 magnetic beads (MiltenyiBiotec, Leiden, The Netherlands) with a MACS® MultiStand and LS Column by passing 3 mL of MACS buffer (2 mM ethylenediaminetetraacetic acid (EDTA) and 0.1% FCS in PBS) according to the manufacturer's protocol.

For maturation into macrophages, monocytes were washed and cultured at a concentration of 2 × 10^6^ cells/mL in Petridishes in macrophage medium (DMEM, Invitrogen, Breda, The Netherlands), supplemented with 5% (v/v) normal human serum (Bio Whittaker, East Rutherford, NJ, USA), and 1% (v/v) penicillin-streptomycin-glutamine (Invitrogen), at 37°C, 5% CO_2_. Monocytes matured into macrophages (M0 macrophages) in the course of 5 days of culturing. After 5 days the supernatant was removed and cells were harvested by adding 4% lidocaine (v/v) in PBS to the Petridish, incubated for 10 minutes, scraped to aid detachment, and counted. Macrophages were washed and reseeded in 6-well plates in a concentration of 10^6^ cells/mL in macrophage medium and cultured for an additional 2 days.

### Macrophage activation

Generation of pro-inflammatory M1 macrophages and anti-inflammatory M2 macrophages was performed as previously described [7,26]. In brief, M1 macrophages were obtained by priming M0 macrophages with 1,000 U/mL IFNγ(U-Cytech, Utrecht, The Netherlands) for 24 h; next, 10 ng/mL *Escheria coli*-derived lipopolysaccharide (LPS) (026:B6; Sigma-Aldrich, Zwijndrecht, The Netherlands) was added to the macrophage medium for a further 24 h. To generate M2 macrophages, 10 ng/mL recombinant human IL-4 (Immunotools, Friesoythe, Germany) was added to the culture medium for 48 h. As a control (M0) macrophages were cultured for 7 days without additional stimuli.

### Cytokine measurements

The production of pro- and anti-inflammatory mediators was assessed by ELISA in cell-free macrophage-conditioned medium sampled 48 h after activation using commercial kits for human IL-4, IL-10, IL-6, and TNF-α (Sanquin blood bank). These cytokines were chosen as they are able to characterize M1 (TNF-α, IL-12) and M2 (IL-10, IL-4) activation status [27,28]. In all experiments, medium containing the activation cytokines (IFNγ and LPS or IL-4) was assessed as well as the supernatants from M0, M1 and M2 with an ELISA. Absorbance was read at 450 nm on a spectrophotometer and unknown sample concentrations were calculated using an equation from the standard curve. Supernatants of three independent experiments were used.

### Migration of macrophages

Directed migration experiments were performed using a TAXIScan (ECI, Tokyo, Japan) [29], consisting of a chip, a metal plate with 12 wells. Two opposing chambers (A, B) are connected by an 8-μm-wide 260-μm-long migration path. Macrophages were injected in the first well (A). To align the cells on to the starting position, 10 μL was ejected from the adjacent well (B). Next, the different chemokines that are known to be upregulated in neuroinflammatory diseases such as MS (CCL2, CCL5, CXCL10 and CXCL12 and the complement factor C1q (2 μL)) (for detailed information see Table 1) were injected into the same well (B). First the optimum concentration of the chemokine was determined; in the TAXIScan M0 and M2 macrophages either migrated to the added chemokine or remained sessile as all M1 cells remained sessile continuously. No bell-shaped curve was observed with increasing concentrations (data not shown). Next, the optimised concentrations were used to study the migration capabilities between M0, M1 and M2. Cellular migration at 37°C was recorded with a camera focussed on the migration path every 30 sec for 1 h. The migration profile was analysed afterwards with manual tracking software from ImageJ with the chemotaxis and migration plug-in [30]. In each experiment, ten macrophages were randomly selected for manually tracking. With this software, the difference in the centre of mass at the beginning and at the end of the experiment was calculated; this value represents the length of migration for cells. The forward migration index (represents the directness towards a definite point of cell trajectories) and velocity of the cells was calculated.

**Table 1:** Chemokines used in migration studies

**Chemokine**	**Dilution**	**Source**
CCL2 (MCP-1)	2 ng/mL	Prepotech, Germany
C1q	1nM	Quindad, USA
CXCL10	10 ng/mL	Quindad, USA
CXCL12	30 ng/mL	RD, UK
CCL5	1 ng/mL	RD, UK

CCL, Chemokine C-C motif ligand; CXCL, Chemokine C-X-C motif ligand.

### Macrophage adhesion

The adhesion of M0, M1 and M2 macrophages to extracellular matrix molecules was determined using plates coated with either collagen type 1 (from calf skin, Sigma Aldrich, Saint Louis, MO, USA) or fibronectin isolated from human plasma (Roche, Woerden, The Netherlands). M0, M1 and M2 macrophages were harvested (as described above) and washed with PBS, labelled for 15 minutes at 37°C with 1 μL (2′,7′bis (2carboxyethyl) 5 (and6) carboxyfluoresce) (BCECF-AM) (Invitrogen). Cells were washed and seeded onto a 96-well plate in a density of 10^5^ cells per well and cultured for 2 h at 37°C and 5% CO_2._ After 2 h, non-adherent cells were removed. The remaining cells were lysed with 0.1 N NaOH and fluorescence was measured in a Fluostar24 (BMG lab technologies, Offenburg, Germany). The percentage of adherent cells was determined by comparing fluorescence intensity to a calibration line ranking from 0 cells to 1.10^6^ cells/mL.

### Chemokine receptor expression

Macrophages were harvested 48 h after activation using PBS containing lidocaine 4% (Sigma Aldrich,) and the supernatants collected for ELISA. Cells were washed twice with PBS and fixed with PBS containing 4% formalin. Subsequently the macrophages were labelled for 1 h at room temperature with primary antibody (Table 2) diluted in PBS containing 0.1% BSA/ 0.1% saponine. Cells were washed twice in PBS and incubated for 1 h (room temperature) with fluorescent-labelled secondary antibody diluted in PBS 0.1% BSA, washed twice and resuspended in fluorescence-activated cell sorting (FACS) buffer prior to analysis. Four-colour flow cytometry (FACSCalibur, Becton Dickinson, Erembodegem, Belgium) was used in combination with Cell Quest software (Becton Dickinson) and FlowJo software version 9.4.0 for Microsoft (Tree Star Inc, Ashland, OR, USA) to analyse expression of markers (see Table 2) on the differentially activated macrophages. As controls the primary antibody was replaced by an isotype control (Table 2). The cells were stained with a viability dye according to the manufacturer's instructions (Fixable Viability Dye eFluor 660, eBioscience Hatfield, United Kingdom) at a concentration of 1 μL dye per 10^6^ cells. Viable cells were gated (approximately 70%) as described before [7] and the mean fluorescence of each marker was compared to the M0 macrophage expression. For each measurement of markers of M0, M1 and M2 macrophages at least 10,000 cells were gated and analyzed. Each analysis was repeated using cells from three different donors.

**Table 2:** Antibodies used for FACS analysis

**Antigen**	**Species and isotype**	**Clone**	**Dilution**	**Source**
CD31	mIgG1	JC70A	1:100	Dako, NL
VLA-4	mIgG1	HP2/1	1:100	Millipore, NL
CCR2	rbIgG	E68	1:50	Abcam, UK
CXCR3	mIgG1	1C6	1:50	BD, NL
CXCR4	mIgG1	12G5	1:50	BD, NL
CCR5	mIgG1	2D7	1:50	BD, NL
Isotype control	mIgG1	-	0.2 μg/mL	Serotec, UK

Rb, rabbit; m, mouse.

### Cytoskeleton visualization

To analyse the cytoskeleton of the macrophages, the actin cytoskeleton was visualized. Macrophages were harvested after 5 days culture, reseeded on glass coverslips and then activated with IFNγ/LPS or IL-4 to induce M1 or M2 subsets. M0 macrophages were cultured in medium without stimuli. After 48 h, cells were left untreated or activated with CCL2 10 μM (Peprotech, Heerhugowaard, The Netherlands) for 10 minutes fixed with 4% paraformaldehyde in PBS for 30 minutes at 4°C, rinsed with PBS and permeabilized with 0.2% Triton X-100 in PBS for 5 minutes. The cells were rinsed with 10 mM Glycine, rinsed with PBS, and incubated with Cdc42 (1:500 Abcam, Cambridge, UK) for 1 h. The cells were rinsed with PBS prior to staining with the second antibody goat anti rabbit ALEXA 488 (1:400, Invitrogen) and rhodamine phalloidin (1:300, Sigma Aldrich) a high affinity, fluorescent filamentous actin probe for 1 h [9]. Cells were washed twice and the nuclei counterstained using Hoechst (1:5000, Sigma Aldrich) and washed again and mounted in mounting medium (88% hydrolysed polyvinyl alcohol, Dako, Heverlee, Belgium). Images were captured on a Leica DM6000 microscope.

### Statistical analysis

Statistical analysis was performed using Graphpad Prism version 4.03 for Windows (Graphpad software, San Diego, CA, USA). The FACS analysis, morphology, adhesion and migration experiments were analysed using one-way analysis of variance (ANOVA) with the Bonferroni correction. The results were considered statistically significant when *P* was <0.05.

## Results

To investigate the migration capacities of differently activated macrophages, we examined the rate of migration towards a chemoattractant, their ability to adhere to different extracellular matrices, the differential expression of chemokine receptors and changes in the cytoskeleton.

### Macrophage activation

**Figure 1:**
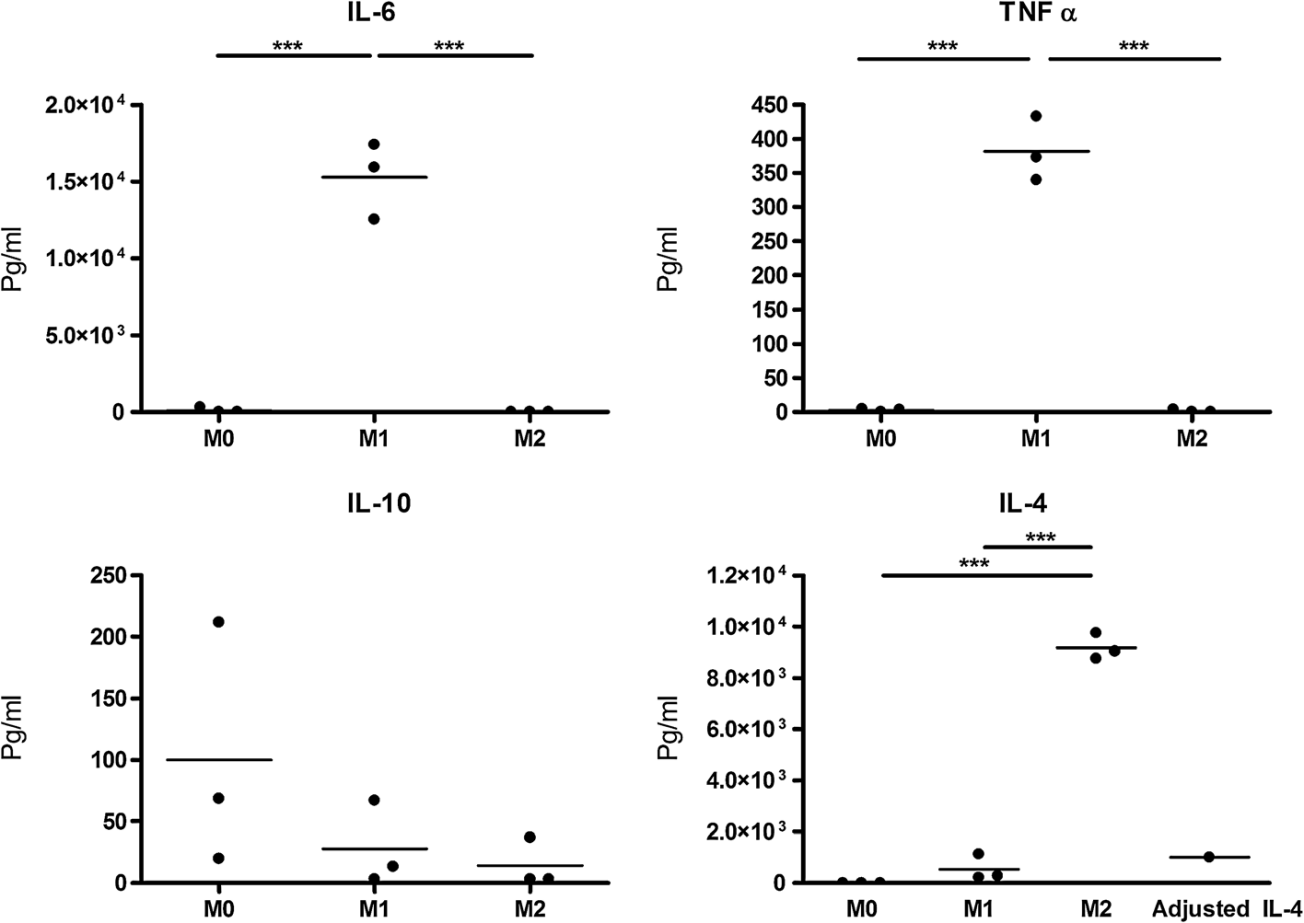
**Cytokine profile of M0, M1 and M2 macrophages.** Cytokine profile of the differently activated macrophages measured with ELISA. The M1 macrophages produce more pro-inflammatory cytokines IL-6 and TNF-α; the M2 macrophages produce more IL-4 and negligible levels of IL-10. The experiments were performed on three different donors.

First the effect of M1 and M2 induction was assessed by determining their cytokine profile. The cytokines measured in the supernatants for M1 polarised cells showed a significant increase of IL-6 and TNF-α compared to M0 (Figure 1). In contrast, M2 macrophages showed a significant increase in levels of IL-4 (corrected for the stimulation with IL-4); IL-10 was produced by all subsets at negligible levels. Taken together, the cytokine profile confirmed the M1 and M2 activation status of the macrophages (Figure 1).

### Migration

**Figure 2:**
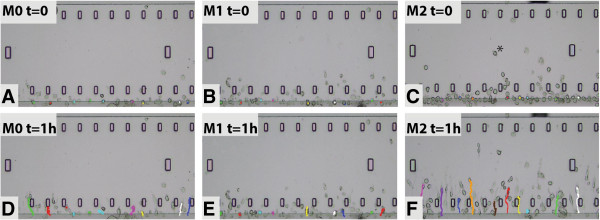
**Migration of M0, M1 and M2 macrophages in a TAXIScan.** Representative pictures of a migration experiment in the TAXIScan. **(A-C)** Pictures at time (t) point zero (the cells that crossed the line have not migrated (*)), with all the cells lined up for migration. The cells tracked with ImageJ are visualized with dots. **(D-F)** Pictures taken after 1 h: lines represent the migration paths of the macrophages; **F** clearly shows that the M2 macrophages migrated further as indicated by the lines compared to M0 and M1.

**Figure 3:**
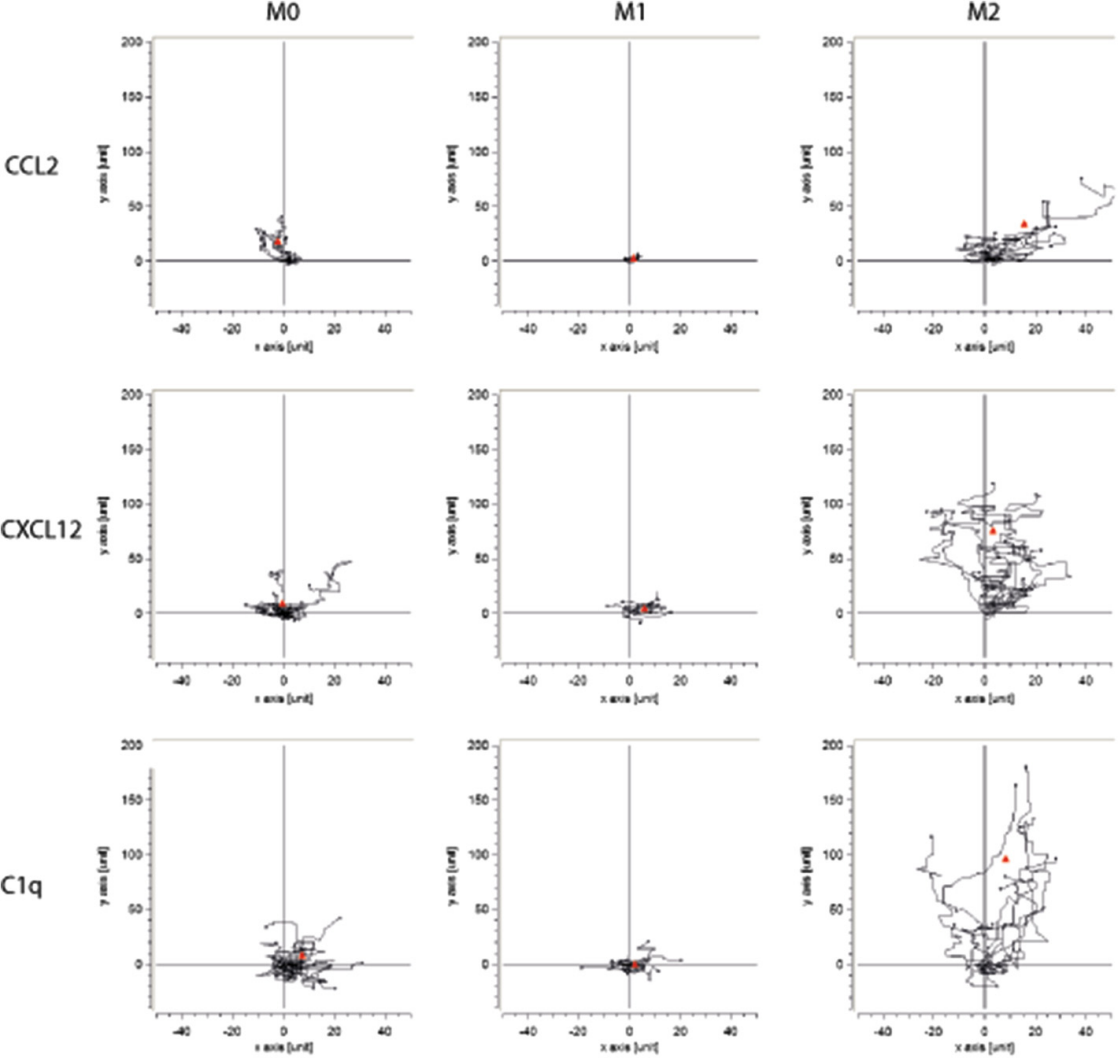
**Two-dimensional trajectory plots of M0, M1 and M2 for CCL2, CXCL12 and C1q.** Representative figures of the directed cell migration in the TAXIScan; the upper panel shows migration tracks of M0, M1 and M2. The centre of mass is depicted with a red triangle, M2 and M0 migrate comparable towards CCL2. The middle panel shows migration of M0, M1 and M2 towards CXCL12; M2 migrates over larger distances than M0 and M2. C1q is the most powerful attractant for M2 macrophages, as represented in the lower panel. CCL, Chemokine C-C motif ligand; CXCL, Chemokine C-X-C motif ligand.

**Figure 4:**
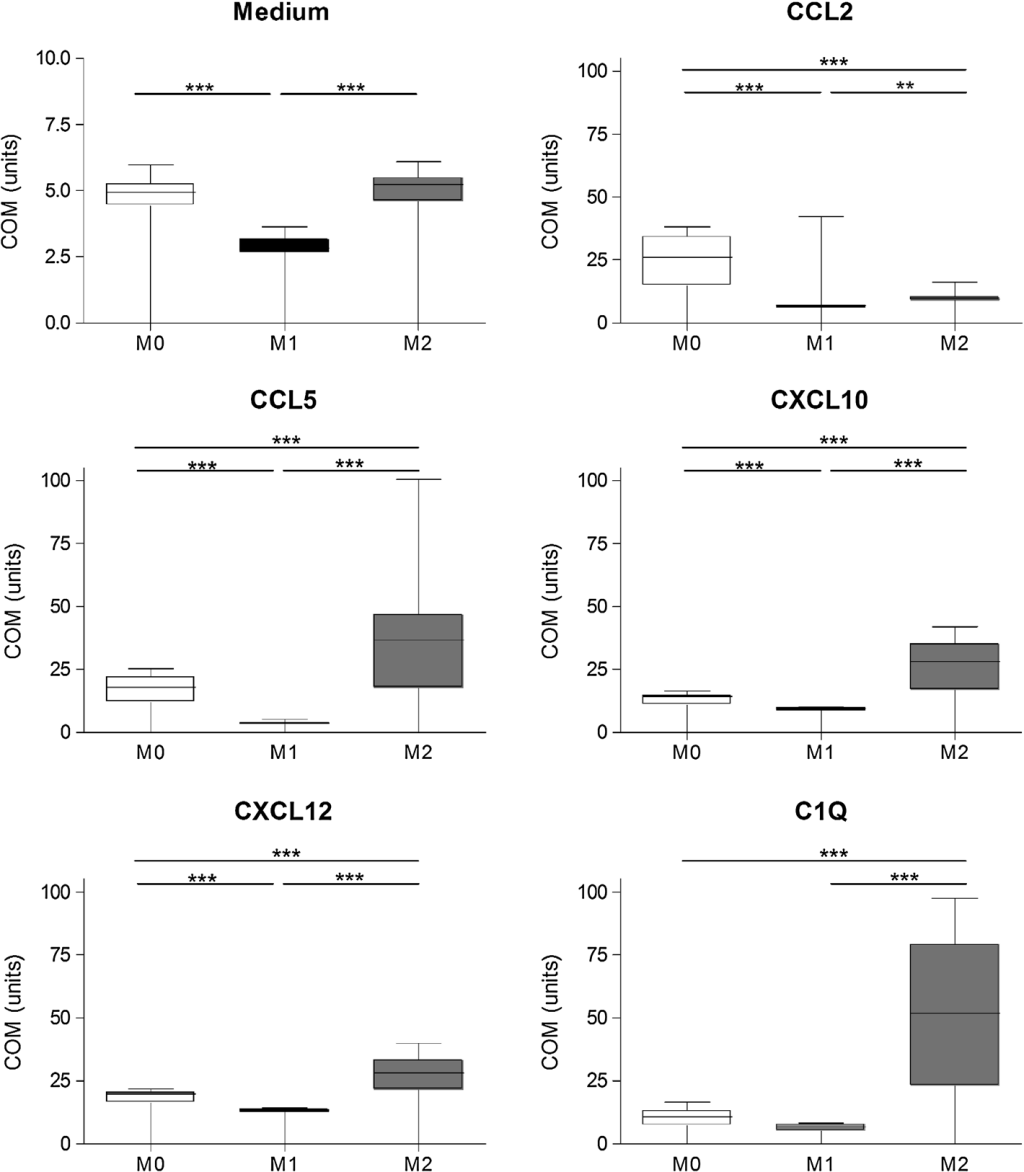
**Quantification of centre of mass of the migrated macrophages.** The centre of mass (COM) as measurement for migration of 10 tracked cells of three independent experiments towards chemokines and complement factors. M2 macrophages consistently migrate larger distances than M1 macrophages. Only to CCL2, the M0 macrophages migrate distances comparable with M2 macrophages. The figures represent the means of three different experiments; **P < 0.01, ***P < 0.001. CCL, Chemokine C-C motif ligand; CXCL, Chemokine C-X-C motif ligand.

Next, we investigated the migratory capacities of M0, M1 and M2 macrophages, by assessing both spontaneous motility and directed migration towards CXCL10, CXCL12, CCL5, CCL2 and C1q. A live cell imaging assay was used to assess directed migration capabilities of M0, M1 and M2 in medium for 1 h. Representative pictures of the migration assays are depicted in Figure 2. Cells were manually tracked using ImageJ and two-dimensional trajectory plots for CCL2, CXCL12 and C1q are shown in Figure 3. M2 macrophages were found to migrate over longer distances and in a more direct fashion compared to M1. For almost all chemoattractants studied, M2 migrated over significantly longer distances than M0 and M1 macrophages, as represented as centre of mass (Figure 4). M0 macrophages migrated over a distance that was intermediate between M1 and M2 macrophages, except towards CCL2. M2 macrophages show enhanced velocity compared to M0 and M2 dependent on the stimuli used (Additional file 1: Figure S1). Directionality of the cells is used to characterize straightness of migration from the starting point to the endpoint. In the presence of CCL2, CXCL10 and C1q, M2 macrophages have a more direct migration (as indicated with forward migration index (FMI) tract compared to M0 macrophages (Additional file 2: Figure S2). The M1 macrophages show an enhanced direct tract upon attractant C1q compared to M0 macrophages. Representative movies of the migration studies are available in Additional files 3, 4 and 5.

### No differences in adhesion between M0, M1 and M2 macrophages

**Figure 5:**
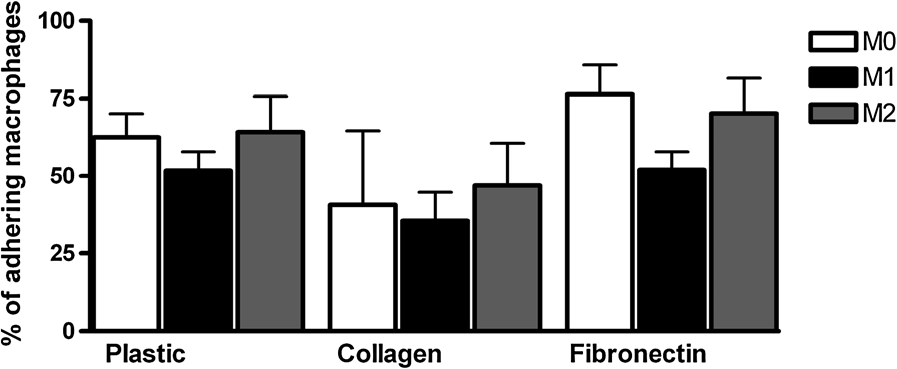
**Adhesion capacities of M0, M1 and M2 macrophages.** The adhesion capabilities represented in percentage of adhesion of the differently activated macrophages after two hours of incubation on uncoated plates, or plates coated with either fibronectin or collagen. The grey bars depict the M2 macrophages, the black bars the M1 macrophages and the empty bars represent M0 macrophages. Data are the mean and standard error of the mean of n = 3.

**Figure 6:**
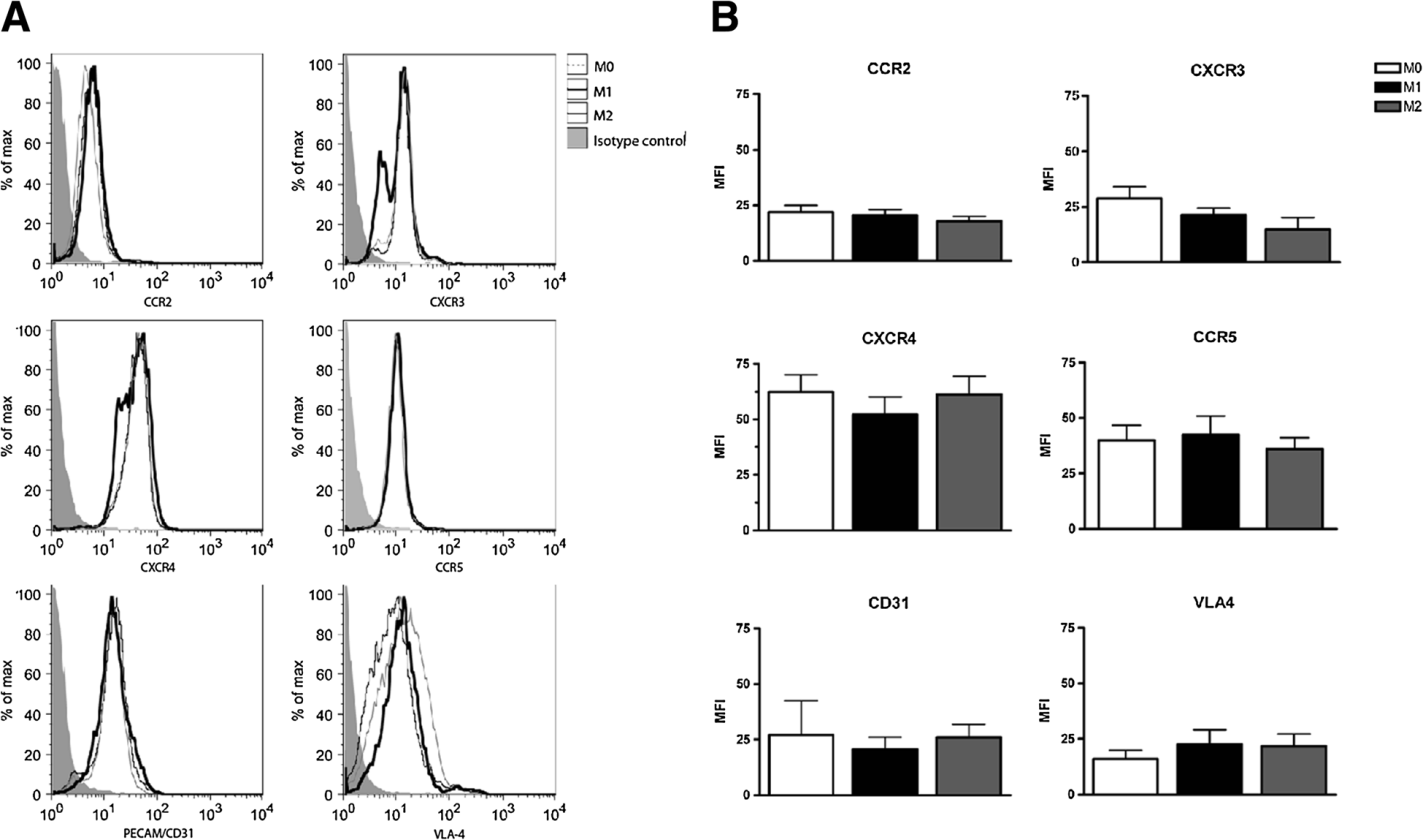
**Analysis of adhesion molecules and chemokine receptors.** Fluorescence-activated cell scan (FACS) plots of the adhesion molecules and chemokine receptors between the subsets. **(A)** The isotype control is depicted in the filled line, M0 is the thin black line, the bold black line represents M1, and the grey line M2. **(B)** Mean fluorescence intensity (MFI) of expression of very late antigen-4 (VLA-4) and platelet endothelial cell adhesion molecule (PECAM/CD31) between the subsets. Chemokine receptor expression using FACS presented in MFI for the different subsets of macrophages. The histogram are representative of three donors and the MFI figures are means and standard error of the mean of three different experiments. CCL, Chemokine C-C motif ligand; CCR, receptor for CCL; CXCL, Chemokine C-X-C motif ligand; CXCR, receptor of CXCL.

To determine the potential differences in the adhesion capacity of M0, M1 and M2, the adhesion to different extracellular matrices was assessed. No significant differences in adhesion to uncoated plastic, collagen or fibronectin coated wells were observed between M0, M1, and M2 macrophages (Figure 5). Analysis of the expression of the molecules that mediate cellular adhesion to collagen and fibronectin, platelet endothelial cell adhesion molecule (PECAM/CD31) and integrin α4β1 (very late antigen-4 (VLA-4) revealed no differences between the differently activated macrophages (Figure 6).

### Chemokine receptor expression

Next, we analysed the surface expression of CCR2, CCR5, CXCR4 and CXCR3, which are receptors for CCL5, CCL2, CXCL12 and CXCL10, respectively. Our studies showed that the chemokine receptor expression did not differ between M0, M1 or M2 macrophage subsets (Figure 6).

### Differential cytoskeletal rearrangements of M2 vs. M1

**Figure 7:**
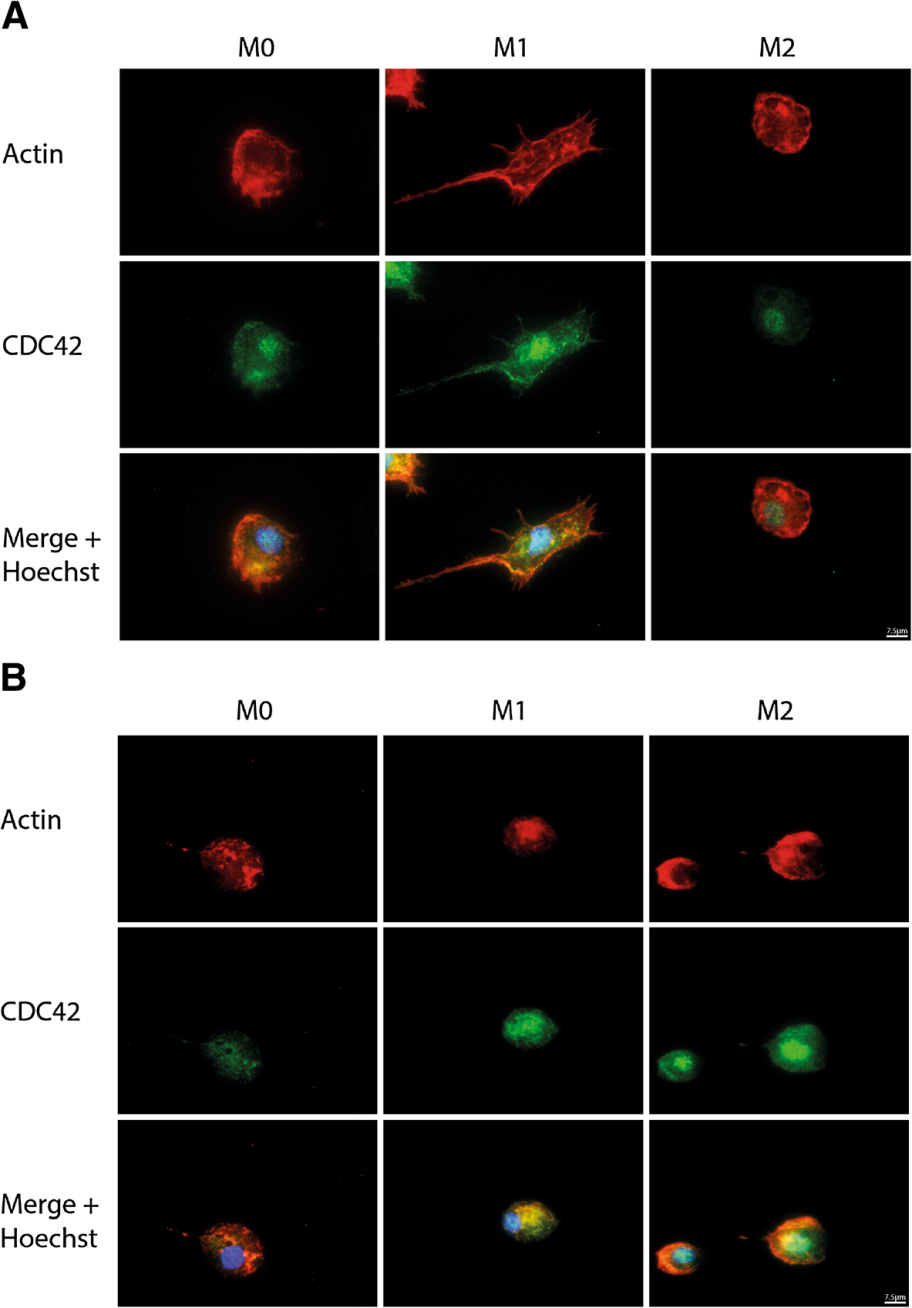
**Macrophage activation affects cytoskeleton.** Differences in morphology of differently activated macrophages visualised by actin organization (red) Cdc42 distribution (green) and a nuclear stain Hoechst (blue). **(A)** The M1 macrophages have the actin concentrated at the cell cortex and the Cdc42 is randomly distributed throughout the cell. M1 macrophages are more elongated than the M2, the actin in M2 is more diffusely distributed throughout the cell compared to M1, and Cdc42 is restricted to the nucleus. Control macrophages have a morphology in between these polar ends and the Cdc42 is present in the nucleus as well as in the cytosol **(B)**. After stimulation with CCL2 for 10 minutes the cytoskeleton rapidly changes, and all activated macrophages adopt a spherical appearance. In M1 macrophages the Cdc42 distribution remains in the cytosol, the M0 macrophages have the Cdc42 co-localizing with actin in the cytosol. M0 and M2 macrophages develop filopodia and the Cdc42 co-localizes with the actin **(B)**.

Next, we determined the cytoskeleton rearrangements of the different macrophage subsets by staining cellular F-actin with phalloidin. Striking differences were found in the organization of the cytoskeleton between M0, M1 and M2 macrophages. Untreated macrophages (M0) showed a mixture of round and spindle-shaped cells in which actin was diffusely distributed throughout the cell and partially concentrated in the cell cortex. Upon treatment with IFNy and LPS (M1), macrophages became spindle-shaped, in which the actin fibres were randomly spread throughout the cell, with clusters at the cell cortex. After stimulation with IL-4, M2 macrophages were mostly spherical and displayed actin condensed at protrusions. Cdc42, relevant for making filopodia, was randomly distributed through M1 and in the nucleus, whereas in M2 macrophages it was only present at the nucleus (Figure 7A).

To further investigate the role of the cytoskeleton in the capability of migration of the macrophages, M0, M1 and M2 macrophages were activated with CCL2, upon which M0, M1 and M2 macrophages adopted a spherical morphology and the cytoskeleton was rapidly rearranged. M0 macrophages showed no co-localization of actin and Cdc42, and formed filopodia. In M2 macrophages, the actin formed a dense network and was co-localizing with Cdc42 at the part where filopodia are formed. M1 macrophages showed a diffuse distribution of actin which showed almost complete co-localization with Cdc42 (Figure 7B).

## Discussion

In neuroinflammatory diseases like MS, macrophages play a dual role. Depending on their activation status, they are considered to play either a detrimental (neuron) damaging, pro-inflammatory (M1) or neuroprotective, anti-inflammatory (M2) role, as revealed in experimental autoimmune encephalomyelitis (EAE, an animal model for MS) and a spinal cord injury model [1,4,31]. The effect of the activation status on migratory properties of human macrophages has not been studied yet and may differ from that observed in the rodent situation. Therefore, the aim of this study was to reveal the functional differences in terms of, migration, adhesion and adhesion molecules and chemokine receptor expression upon M1 and M2 activation. Our findings indicate that anti-inflammatory M2 macrophages migrate over longer distances and with higher velocity towards CCL5, CXCL10, CXCL12 and C1q compared to sessile M1 macrophages. In addition, M0 and M2 macrophages are more adapted to rearrange their cytoskeleton upon activation with CCL2 by making filopodia, whereas M1 macrophages adapt a spherical morphology.

M2 macrophages migrated over longer distances towards chemoattractants CCL5, CXCL10, CXCL12 and C1q, whereas M1 macrophages do not respond and remain sessile. The un-activated macrophages (M0) show an intermediate migration capability. The migration of M0 exceeds M1 and M2 towards chemokine CCL2, one of the key factors for monocyte recruitment into MS lesions [23,32]. Our results are partly in line with our previous studies on murine macrophages where the migration of M2 macrophages to CXCL12 and CCL5 was enhanced compared to M1 macrophages [9]. However, murine macrophages revealed no differences between the M0, M1 and M2 migration capabilities towards CCL2.

In our study C1q is the most potent attractant for M2 macrophages; interestingly, it was recently shown that macrophages show an anti-inflammatory phenotype upon activation with C1q [33]. The same group showed that C1q itself is neuroprotective [34]. Our new finding that C1q predominantly attracts M2 could be an additional beneficial mechanism for promoting repair in the CNS [1,8,31].

To understand the altered migration between the different subsets of macrophages, the adhesion to different extracellular matrix components was studied as well as the expression of integrins and adhesion molecules [35-38]. In contrast to our findings in murine cells [9], here we observed no differences in cellular adhesion between human un-stimulated, M1- and M2-activated macrophages to various substrates, indicating that the adhesive capacities play no role in our observed differences in motility and migration. Also no significant differences were observed in the expression of the adhesion molecules PECAM/CD31 and VLA-4 on M0, M1 and M2 macrophages. To elucidate the potential role of chemokine receptor expression upon activation of the macrophages, FACS analysis of the chemokine receptor expression was performed, again showing no significant differences between M0, M1 and M2.

However, we were able to show a clear difference in morphology and cytoskeletal arrangement between M1 and M2 macrophages, which may underlie the altered migration capacity of the different subsets. M1 macrophages are elongated, whereas M2 macrophages have a more circular appearance. M1 macrophages have a dense static actin-network along the cortex, which could explain the limited migration, whereas the M2 macrophages have more randomly distributed actin. Differences in morphology and migration is known to be dependent on changes in the cytoskeleton and the actin reorganization, a process regulated by small guanine triphosphate (GTP)-binding proteins (Rho, Rac and Cdc42) [34,39-43]. Little is known about the role of the cytoskeleton with regard to the activation status of human macrophages. IFNγ is known to induce polymerisation of actin fibres, a process known to influence morphology but not migration capacities [43,44]. In our study we used IFNγ/LPS and IL-4, respectively, to induce M1 and M2 macrophages. Our findings are in line with Porcheray *et al*. who described that IL-4 activated human macrophages adopted a spherical morphology and activation with IFNγ without the additional treatment of LPS stimulated elongated morphology [22]. Our data differ from our earlier observations using murine bone marrow-derived macrophages, where the M1 (IFNγ- and LPS-induced) macrophages were more circular and the M2 macrophages (IL-4-induced) elongated [9]. Upon stimulation with CCL2, M0 and M2 macrophages were able to form filopodia, and Cdc42 co-localized with actin, indicating that both M0 and M2 can be polarised for migration, whereas M1 are not. This is the first step in unravelling the differences in the migration capabilities of M1 and M2 macrophages in humans. The exact working mechanism of IFNγ, LPS and IL-4 on the cytoskeleton needs further investigation. Other possible mechanisms that would explain the differences between the migration properties of M0, M1 and M2 could be dependent on differences in ATP [45] or Ca2+ homeostasis [46].

In summary, our studies reveal substantial differences in the migratory capabilities of M1 versus M2 human macrophages, and that M2 macrophages are more motile and migrate over longer distances towards chemoattractants involved in CNS inflammation. In contrast, macrophages in a pro-inflammatory state, are sessile and do not migrate towards these chemoattractants. M2 macrophages are known to be growth-promoting and are able to secrete neurotrophic factors. Further elucidation of macrophage migration across a variety of tissues holds great potential for understanding the role of M1 and M2 macrophages in neuroinflammatory diseases such as MS.

## Abbreviations

AA/M2: Alternatively activated; BCECF: 2′ 7′′-bis-(2-carboxyethyl)-5-(and-6)-carboxyfluorescein; BSA: Bovine serum albumin; CA/M1: Classically activated; CCL: C-C motif chemokine ligand; CCR: Receptor for CCL; CNS: Central nervous system; COM: Centre of mass; CXCL: C-X-C motif chemokine ligand; CXCR: Receptor for CXCL; DMEM: Dulbecco’s modified Eagle's medium; EAE: Experimental autoimmune encephalomyelitis; EDTA: Ethylenediaminetetraacetic acid; ELISA: Enzyme-linked immunosorbent assay; FACS: Fluorescence-activated cell scan; FCS: Fetal calf serum; FMI: Forward migration index; IFN-γ: Interferon-γ; IL: Interleukin; LPS: Lipopolysaccharide; MFI: Mean fluorescence intensity; MS: Multiple sclerosis; PBMC: Peripheral blood mononuclear cells; PBS: Phosphate-buffered saline; PECAM/CD31: Platelet endothelial cell adhesion molecule; TNF: Tumour necrosis factor; VLA-4: Very late antigen-4.

## Competing interests

The authors declare that they have no competing interests.

## Authors’ contributions

DV was involved in acquisition of data, data analysis and writing of the manuscript. PH, MB performed both data acquisition and analysis. AT and HV were involved in data acquisition and interpretation. SA and CD participated in study design, conceptualization, data analysis and helped to draft the manuscript. All authors read and approved the final manuscript.

## Supplementary Material

Additional file 1: Figure S1 Quantification of velocity and directness of the macrophage subsets. The velocity of the macrophages in the migration assay was calculated with ImageJ. M2 macrophages have a significantly higher velocity towards CCL5, CXCL10, CXCL12 and C1q compared to M1. M0 have a higher velocity towards CXCL10 and CXCL12 than M1. Towards CCL2 M0 macrophages migrate faster than M2. CCL, Chemokine C-C motif ligand; CXCL, Chemokine C-X-C motif ligand. Click here for file

Additional file 2: Figure S2 Quantification of forward migration index. The quantification of the forward migration index of the macrophages in the migration assay was calculated with ImageJ. M2 macrophages have a significantly higher forward migration index towards CXCL10, CXCL12 and C1q compared to M1. Towards CCL2 M0 macrophages exceed M2 macrophages. CCL, Chemokine C-C motif ligand; CXCL, Chemokine C-X-C motif ligand. Click here for file

Additional file 3: Movie 1 M0 macrophages migrating towards CXCL12. A few of the M0 macrophages lined up under the horizontal line are migrating towards the CXCL12. CXCL, Chemokine C-X-C motif ligand. Click here for file

Additional file 4: Movie 2 M1 macrophages migrating towards CXCL12. None of the M1 macrophages lined up under the horizontal line are migrating towards the CXCL12. The cells are able to transform their cell body, however fail to migrate towards the CXCL12. CXCL, Chemokine C-X-C motif ligand. Click here for file

Additional file 5: Movie 3 M2 macrophages migrating towards CXCL12. The majority of M2 macrophages lined up under the horizontal line are migrating towards the CXCL12. These cells show high capacity to transform their cell body migrate towards the CXCL12. CXCL, Chemokine C-X-C motif ligand. Click here for file
