# Supplementary figures and images for: Macrophages migrate in an activation-dependent manner to chemokines involved in neuroinflammation
Source: J Neuroinflammation. 2014 Feb 1;11:23. doi: 10.1186/1742-2094-11-23 (PMC3937114; doi:10.1186/1742-2094-11-23)

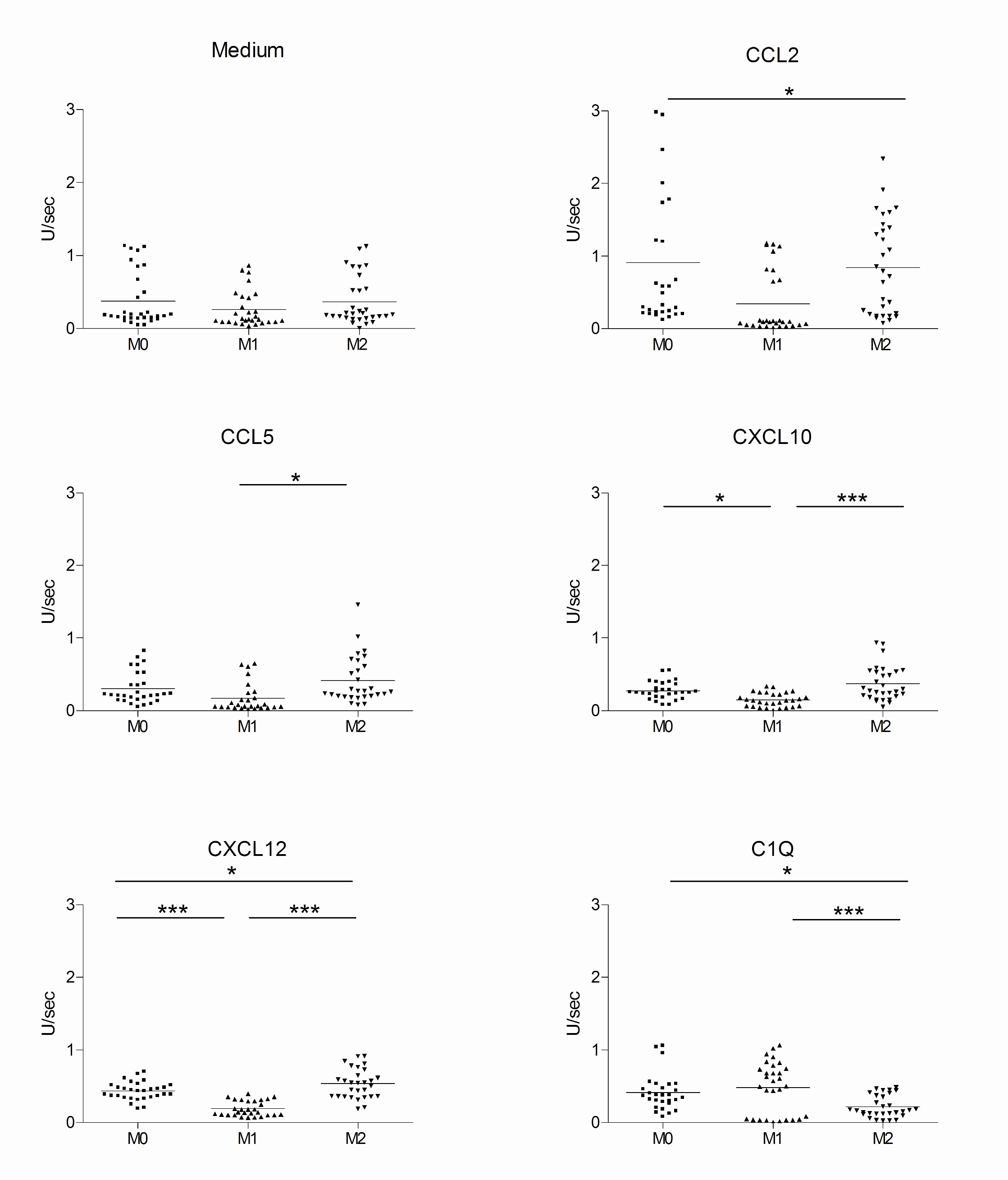

Supplement: Additional file 1: Figure S1 — Quantification of velocity and directness of the macrophage subsets. The velocity of the macrophages in the migration assay was calculated with ImageJ. M2 macrophages have a significantly higher velocity towards CCL5, CXCL10, CXCL12 and C1q compared to M1. M0 have a higher velocity towards CXCL10 and CXCL12 than M1. Towards CCL2 M0 macrophages migrate faster than M2. CCL, Chemokine C-C motif ligand; CXCL, Chemokine C-X-C motif ligand. [file 1742-2094-11-23-S1.tiff]

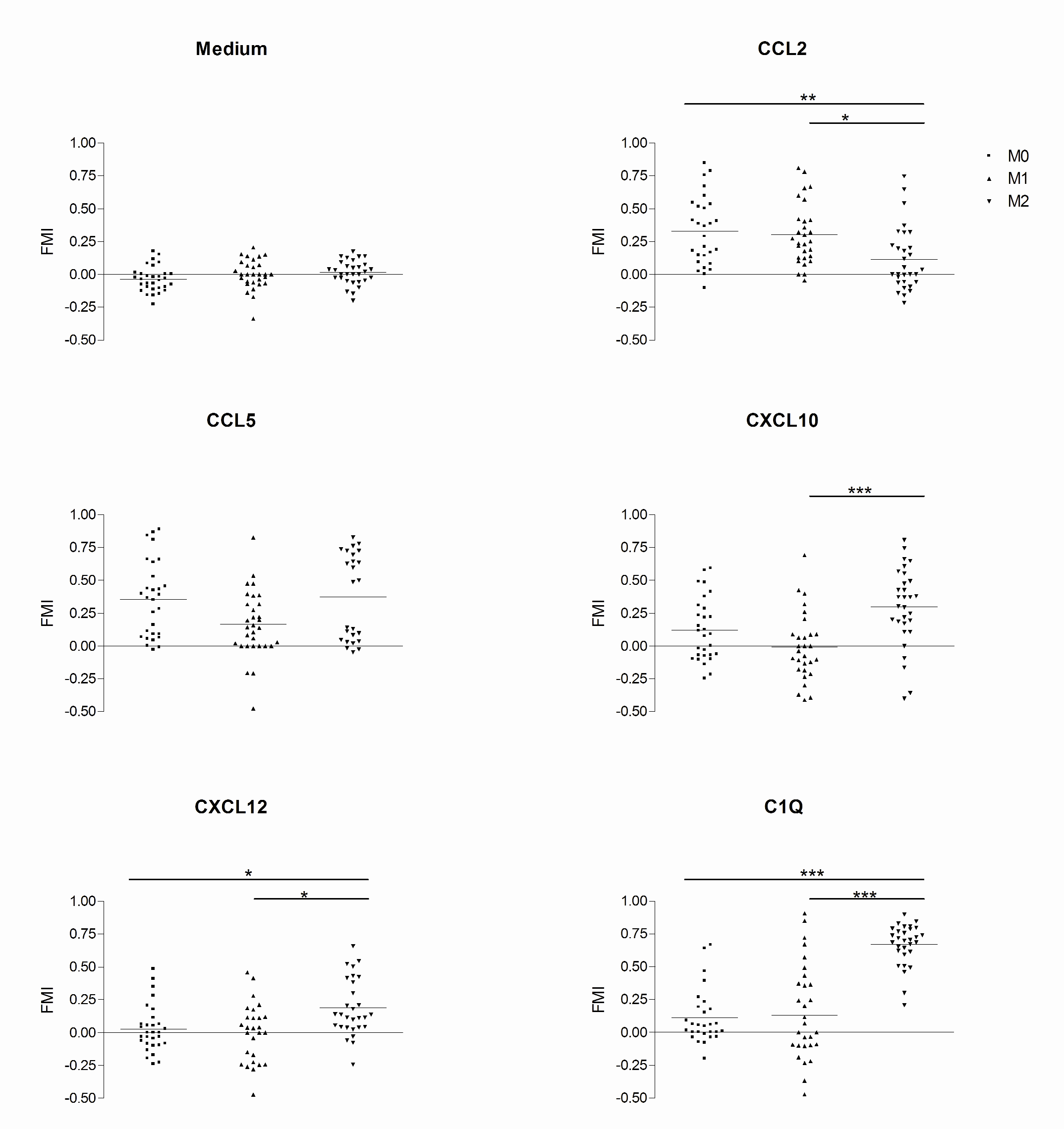

Supplement: Additional file 2: Figure S2 — Quantification of forward migration index. The quantification of the forward migration index of the macrophages in the migration assay was calculated with ImageJ. M2 macrophages have a significantly higher forward migration index towards CXCL10, CXCL12 and C1q compared to M1. Towards CCL2 M0 macrophages exceed M2 macrophages. CCL, Chemokine C-C motif ligand; CXCL, Chemokine C-X-C motif ligand. [file 1742-2094-11-23-S2.tiff]
